# Training in ChiRunning to reduce blood pressure: a randomized controlled pilot study

**DOI:** 10.1186/s12906-015-0895-x

**Published:** 2015-10-15

**Authors:** Kelly McDermott, Deepak Kumar, Veronica Goldman, Haojun Feng, Wolf Mehling, Judith T. Moskowitz, Richard B. Souza, Frederick M. Hecht

**Affiliations:** Osher Center for Integrative Medicine, University of California San Francisco (UCSF), 1545 Divisadero St., 3rd Floor, San Francisco, CA 94115 USA; Department of Radiology and Biomedical Imaging, UCSF, San Francisco, CA USA; Department of Medical Social Sciences, Northwestern University Feinberg School of Medicine, Chicago, IL USA; Department of Physical Therapy and Rehabilitation Science, UCSF, San Francisco, CA USA

## Abstract

**Background:**

People with prehypertension (120–130/80–90 mmHg) are at increased risk of progressing to hypertension. Recommendations for prehypertension include engaging in regular physical activity. We aimed to assess feasibility and acceptability and collect preliminary outcome data on ChiRunning for people with elevated blood pressure. ChiRunning is a commercially available running program based on the mindful movements of Tai Chi, which is aimed at decreasing injury by both increasing body awareness and modifying running form.

**Methods:**

We enrolled adults with elevated systolic (130–150 mmHg) or diastolic (80–100 mmHg) blood pressure in a 12-week pilot trial. Participants were randomized 2:1:1 to 8 weeks of: 1) intervention—a trainer-led ChiRunning group (n = 10); 2) active control—a trainer-led running group (n = 6); or 3) educational control—a self-directed running group (n = 6) and followed for 4 more weeks. The active control and educational control groups were combined for analysis.

**Results:**

This study was feasible, meeting recruitment, retention and adherence goals, and acceptable to participants. Systolic and diastolic blood pressure did not change significantly over the study for either the ChiRunning or control groups. Changes in BMI over time were significantly different from zero in the ChiRunning group (*p* = 0.04) but not in the control group (slope for ChiRunning −0.05 [−0.1 to −0.002] vs. control −0.01 [−0.06 to 0.04], between slope difference, *p* = 0.22). Self-reported running-related *injury* (i.e. discomfort leading to a decrease in running) was similar between groups (ChiRunning, 4 [1.2 to 8.4] vs. control, 3 [0.7 to 7.1] injuries per 100 h of running, *p* = 0.72) although self-reported running-related *discomfort* (i.e. discomfort that does not lead to changes in running) trended higher in the ChiRunning group (ChiRunning, 10 [5.4 to 16.8] vs. control, 4 [1.5 to 9] reports of discomfort per 100 h of running, *p* = 0.06).

**Conclusion:**

ChiRunning appears to be a feasible and acceptable exercise program for people with elevated blood pressure. We did not find that ChiRunning had a significant impact on blood pressure or self reported injury, but did see a positive change in BMI over time. ChiRunning warrants further investigation in a larger trial.

**Trial registration:**

ClinicalTrials.gov Identifier: NCT01587183

## Background

High blood pressure, known as hypertension (≥140/90 mmHg), is a significant problem affecting one in three adults and was associated with 18.8 deaths per 1000 people in the United States in 2010 [1]. Prehypertension (120/80–139/89 mmHg) affects a similar number of people (30 % of adults in the US) and puts individuals at high risk of hypertension [2]. In the Framingham Heart Study, researchers found that 37.3 % of adults <65 years with prehypertensive blood pressure between 130–139/80–89 mmHg developed hypertension in 4 years [3]. Regular physical activity is a recommended treatment for prehypertension [4]. Fitness is inversely related to all-cause mortality [5] and a significant predictor of progression from normo- and prehypertension to hypertension [6, 7]. A recent longitudinal study suggests that fitness also delays typical age-related hypertension [8]. Body mass index (BMI) and waist circumference have also been shown to predict progression from prehypertension to hypertension [7, 9].

Running may be a suitable form of exercise for people with prehypertension because it is typically vigorous, and more intensive activities have been associated with more cardiorespiratory fitness compared to less intensive activities [10, 11]. In addition to increased fitness, running has other benefits including that it is cost effective requiring no equipment, it can be performed with or without others, it can be performed in a wide variety of locations, and it is familiar to most people. Despite these reasons, a major drawback of running is that the increased loading on muscles, joints, and tendons can lead to injury [12]. One study found in a survey of 1,811 people, the most common reason for a relapse from exercise was injury [13].

ChiRunning is a commercially available running program based on the mindful movement practices of Tai Chi. ChiRunning aims to prevent new and recurring injuries by decreasing loading forces on the body by teaching key features of running form, such as using a midfoot strike, reducing overstriding, and increasing cadence. Mindfulness and body awareness practices are also taught as part of the ChiRunning curriculum to both help identify early indicators of injury and to increase motivation [14].

According to a recent American Heart Association statement, “alternative” approaches, including those that increase mindfulness and awareness, may be beneficial adjunctive therapies for prehypertension [15]. Mindfulness Based Stress Reduction (MBSR) uses seated meditation, yoga, and body scan to increase awareness and is one such alternative approach. MBSR has been shown to reduce blood pressure in people with prehypertension and may do so by down-regulating the sympathetic nervous system, improving coping with daily and life stress, and improving adherence to lifestyle modifications such as increased regular physical activity [16, 17]. Like MBSR, ChiRunning uses body scan and other similar tools for mindfulness and body awareness skill building and may be an ideal form of exercise for people with prehypertension.

In this pilot study, we sought to establish feasibility and acceptability and to collect preliminary data on outcomes on a ChiRunning intervention versus control in participants at high risk of hypertension. Feasibility measures included meeting recruitment, retention, and adherence goals, and acceptability was determined through semi-structured participant feedback. The preliminary efficacy data that we collected included blood pressure and BMI over 5 time points (weeks 0, 2, 4, 8, and 12) and self-reported incidence of running-related discomfort and injury over the 12 weeks of the study.

## Methods

### Study design

For this randomized controlled pilot study, a statistician not affiliated with the study used computer generated random blocks of numbers and delivered them to study staff in sealed opaque envelops. Participants were randomly assigned to one of the following three groups with a 2:1:1 allocation ratio: 1) intervention—a trainer-led ChiRunning group (n = 10); 2) active control—a trainer-led running group (n = 6); or 3) educational control—a self-directed running group (n = 6). For the purpose of this article, the two control groups were combined to examine the effects of ChiRunning. Participants met for four training sessions (weeks 0, 2, 4, and 8). The study took place June through September 2012.

### Participants and eligibility criteria

We enrolled adults (age 18–70) living in the San Francisco Bay Area with elevated blood pressure. Participants were recruited using flyers and other print and online advertisements. After an extensive phone screen, eligible individuals were invited to an in-person study visit to have their blood pressure measured at the Osher Center for Integrative Medicine (OCIM).

Our initial inclusion criteria were: 1) systolic blood pressure (SBP) in the range of 130-139 mmHg or diastolic blood pressure (DBP) in the range of 80-85 mmHg, 2) BMI ≤30 kg/m^2^, and 3) not currently taking blood pressure medication. Eligibility criteria were expanded and the recruitment period was extended (from 8 to 12 weeks) as the study team found these criteria as being overly restrictive and limiting enrollment. In the revised eligibility criteria: 1) the upper limit of SBP was increased to 150 mmHg and the upper limit of DBP to 100 mmHg, 2) BMI was increased to ≤35 kg/m^2^, and 3) participants currently taking blood pressure medication were permitted with permission of their physician.

Exclusion criteria included: inability to provide informed consent; any history of serious joint or lower limb injury precluding running as a reasonable exercise program; self-reported inability to run continuously for 5 min (required for the gait analysis reported separately); substance or alcohol abuse, mental health, or a medical condition that, in the opinion of investigators, would make it difficult to participate in the group training sessions; contraindications to moderate-intensity exercise; arrhythmia, alcoholism or other condition that makes accurate blood pressure measurement difficult; a diagnosis of diabetes, chronic kidney disease or other condition indicating tighter control of SBP; non-English speaking (group training was given in English); pregnant or planning to get pregnant during the study period; unwillingness or inability to commit to walk/running up to 30 min three times per week; plans to move from the area during the study time period; and currently exercising at vigorous intensity for greater than 90 min per week. The UCSF Committee on Human Research approved this pilot study and all eligible individuals signed a written, informed consent prior to enrollment.

### Intervention

All participants received a pamphlet on the Dietary Approaches to Stop Hypertension (DASH) diet [18] at their first group training session (or study visit for the educational control group). In addition, all participants were given a training diary booklet. The training diary included a schedule that laid out days of the week to run and amounts of time for each run for the duration of the study. In addition, the training diary included questions about discomfort or injury experienced during the run, and space for other notes or information about the run. All groups were instructed to begin using a walk/run approach, alternating intervals of walking and running to gradually increase intensity.

#### ChiRunning

ChiRunning is a manualized technique requiring certification for all trainers. The ChiRunning group met on Sunday mornings with the certified trainer for an initial 4-h session at week 0, and three more times for 2-h training sessions at weeks 2, 4, and 8. The material covered had two primary focuses: 1) learning specific components of running form, and 2) developing mindfulness and body awareness in the context of running. While each session contained some elements of both focuses, the first two training sessions focused on running form, the third on body awareness and mindfulness and the fourth training session was a review. During each training session, participants were video-recorded while running and received individualized coaching feedback on their running form. Participants met at OCIM for the first half of the session (lecture) and walked 2 blocks to a community center gymnasium for the second half (applied). Participants in the ChiRunning group were given the book *ChiRunning: A Revolutionary Approach to Effortless, Injury-Free Running* [14] and a metronome to wear while running to help achieve target running cadence.

#### Control

Participants were randomly assigned to one of two control groups: 1) active control, an attention matched, group based training with a running coach, or 2) self-directed control, which provided extensive written materials on starting a running practice. The active control group met with a running coach (certified USA Track and Field and Road Runners Club of America) on Sunday afternoons for the same durations as the ChiRunning group. The format of each training session was an initial talk on a running-related topic, followed by questions and answers and a group walk/run. Because of the group walk/run, the training sessions were held at various running-appropriate locations around San Francisco. The talks covered running topics including goal setting, pace, mileage, warm-up/cool down, stretching, core strengthening, cross training, hydration and nutrition, and shoes. Study staff was present at training sessions to ensure adherence to the study protocol. The educational control participants received printed materials on the same running topics covered in the active control talks. The two control groups were combined for analyses given that the primary focus of this article is on ChiRunning and due to small cell sizes.

### Outcomes

Our primary outcomes included feasibility, acceptability and preliminary efficacy for the effect of ChiRunning on blood pressure and BMI. We assessed feasibility based on recruitment, retention, and adherence and acceptability based on participant feedback. Feasibility of recruitment was determined by meeting at least 75 % of our original goal of n = 40, feasibility of retention was determined by retaining 75 % of participants enrolled in the study. Adherence was calculated as the percent of runs and time running laid out in the training schedule that was completed by the participant based on their entries in the training diary. Participants were considered adherent if they met training requirements 75 % of the time. Participants were sent surveys to gauge acceptability of content delivery, training structure and coaches during and after the intervention. Preliminary efficacy outcomes included within-group changes and between-group differences in SBP, DBP, and BMI over the course of the study and self-reported running-related discomfort and injury.

### Measurement

Participants in the control group scheduled study visits to OCIM during normal business hours to have their blood pressure taken for all five blood pressure measures. Out of convenience, participants in the ChiRunning group had blood pressure measurements taken before each of the four training sessions on Sunday mornings. The follow-up visit for the ChiRunning group was scheduled during normal business hours. For all blood pressure visits, participants were asked to sit quietly in a room by themselves for 10 min, after which a trained assessor took two blood pressure measurements two minutes apart using a mercury sphygmomanometer [19]. Weight was measured at each visit; height was measured at the first visit only.

Running-related discomfort and injury were based on entries in the training diary. Self-reported discomfort that did not affect the duration or intensity of the run was considered running-related *discomfort* (RRD). Self-reported discomfort that did affect the duration or intensity of the run was considered to be more severe and classified as running-related *injury* (RRI) [20].

### Data analysis

Given that this pilot was to establish feasibility and acceptability and collect preliminary outcome data, our study was not powered to detect small to medium-sized effects. Based on power calculations for an independent samples *t*-test with a two-sided probability of Type 1 error = 5 % and probability of a Type 2 error = 20 %, with our recruited n = 22, we were able to detect a very large between group effect of d ≥ 1.26.

We used paired *t*-tests with equal variances for within-group comparisons and independent samples *t-*test to examine between-group differences. Fisher’s exact test was used to compare categorical data. We used a linear mixed model to estimate the slope (change over time) and to compare slopes between groups. Discomfort and injury incidence were calculated as new RRD or new RRI reported per 100 h of running, and 95 % confidence intervals were calculated based on a Poisson distribution. We considered *p* ≤ 0.05 to be statistically significant, although *p* ≤ 0.1 was considered to be a meaningful trend given the pilot nature of this study. All statistical analyses were performed using Stata 12 Statistical Software: College Station, TX: StataCorp LP.

## Results and discussion

**Fig. 1:**
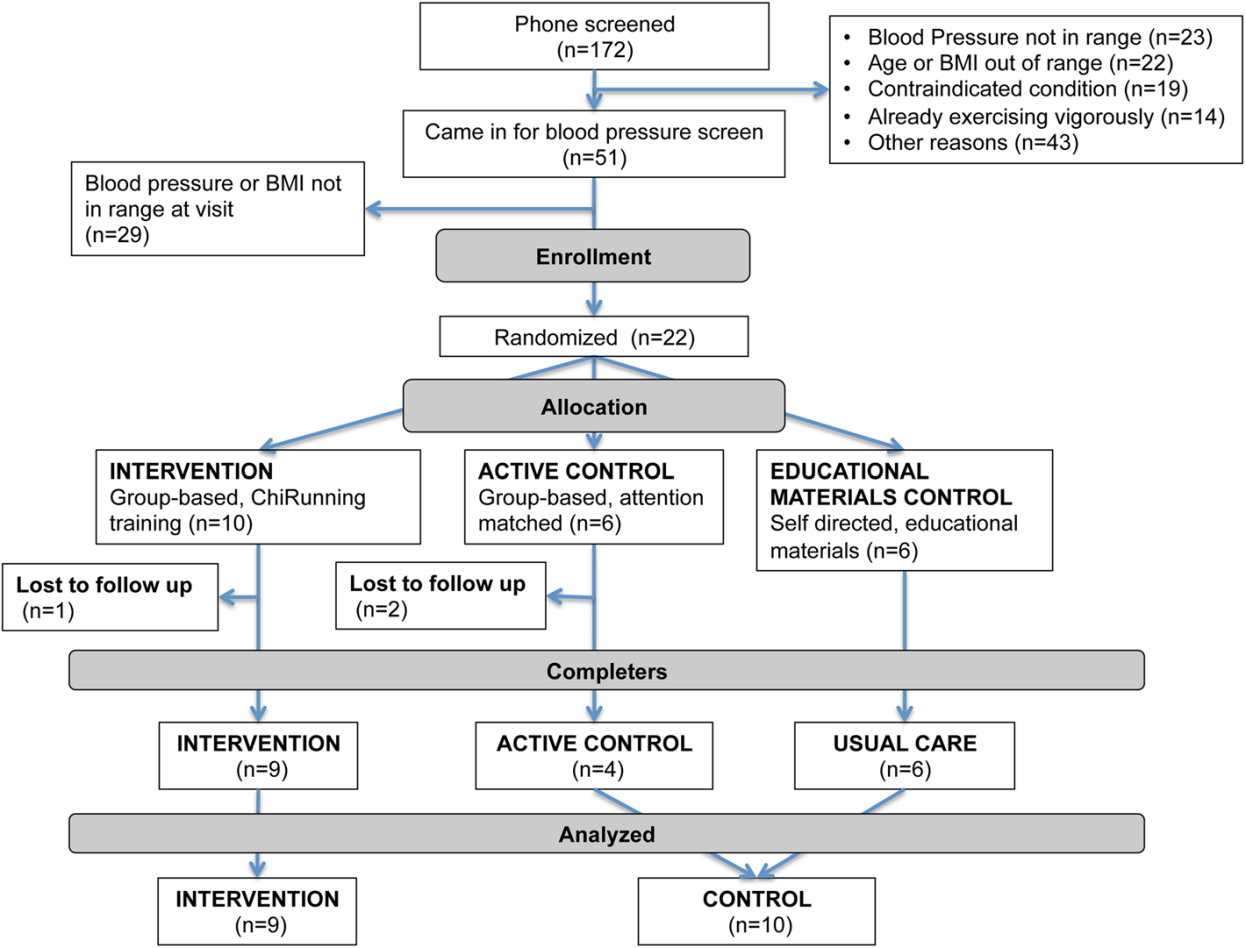
CONSORT flow diagram of participants

Figure 1 illustrates participant flow from screening through data analysis. Half of the 22 enrolled participants (n = 10 ChiRunning, n = 12 control) were female and mean age was 55.4 ± 6.7 years (Table 1). Mean SBP was 137.7 ± 6.7 mmHg, DBP was 87.1 ± 6.0 mmHg and BMI was 26.6 ± 3.1 kg/m^2^.

**Table 1:** Baseline comparison of ChiRunning versus control, mean ± sd unless indicated

	ChiRunning	Control	*P*
	n = 10	n = 12	
Age, mean ± sd	56.4 ± 8.2	54.1 ± 4.5	0.44
Female, n(%)	5(50)	6(50)	0.67
Race, n(%)			
White	7(70)	7(58)	0.66
Black	0	2(17)	
Asian	3(30)	3(25)	
Hispanic ethnicity	0	3(25)	0.22
Systolic blood pressure (mmHg)	136.7 ± 7.0	138.5 ± 6.6	0.53
Diastolic blood pressure (mmHg)	86.6 ± 4.7	87.5 ± 7.2	0.76
Body mass index (kg/m^2^)	26.6 ± 2.8	26.5 ± 3.4	0.94

### Feasibility and acceptability

Based on recruitment, retention, adherence and participant feedback, we determined that the study design and materials were both feasible and acceptable. By revising eligibility criteria we were able to increase recruitment to an acceptable rate. We retained 19 (86 %) participants through study completion. Two participants dropped out after the first training session, one (ChiRunning) for a pre-existing foot problem, and one (active control) due to a scheduling conflict resulting from a new job. Another participant (active control) dropped out after the third training session due to an illness unrelated to running. The three participants that dropped out had baseline measures similar to participants completing the study.

Of the 13 participants in the two trainer-led groups, 11 (92 %) attended at least 3 of 4 training sessions and gave the trainer a mean score of 4 out of 5 for the quality of the teaching. Of the 19 participants completing the study, 14 (73 %) completed at least 75 % of the scheduled runs and 17 (89 %) submitted a training diary that was at least 75 % complete. Participants completed an average of 89 % of the scheduled runs in both groups, and 85 % and 80 % of scheduled time in the ChiRunning and control groups, respectively (*p* = 0.78). Sixteen of the 18 (89 %) responding participants felt they received the tools necessary to maintain their running practice and 14 (78 %) thought that their blood pressure had improved or would improve based on the training they had received as part of the study.

### Blood pressure

There were no significant changes in either group between baseline (week 0) and post intervention (week 8), or between baseline and follow-up (week 12) (Table 2). Neither group’s slope of change over time is statistically different from zero. The change over time appears to be in the right direction in the control group but not in the ChiRunning group at the follow-up measurement (Table 2). This may have been due to the fact that measurements were taken at different times of day according to group. Control participants scheduled all five study visits during normal business hours while ChiRunning participants had the first four measurements taken on Sunday morning before each training session. The last follow up measurement for the ChiRunning group was scheduled during normal business hours because there was no training session. The difference in timing likely introduced a systematic difference in blood pressure between groups, attenuating the first four measurements in the ChiRunning group.

**Table 2:** Within group changes over time and between group differences between ChiRunning and control

	Bl-8wk	*P*	BL - 12wk	*P*	Change over time^b^	*P*
	(post-intervention)^a^		(follow-up)^a^			
Systolic blood pressure (mmHg)						
Within ChiRunning change	−0.22 [−7.81 to 7.37]	0.948	4.33 [−3.1 to 11.77]	0.216	0.3 [−0.2 to 0.8]	0.244
Within Control change	−1.7 [−7.2 to 3.8]	0.502	−2.5 [−7.85 to 2.85]	0.318	−0.21 [−0.68 to 0.27]	0.396
Between group difference	1.48 [−7.04 to 9.99]	0.719	6.83 [−1.48 to 15.15]	0.101	−0.5 [−1.19 to 0.19]	0.153
Diastolic blood pressure (mmHg)						
Within ChiRunning change	−4.44 [−10.78 to 1.9]	0.145	−0.67 [−7.23 to 5.9]	0.821	−0.01 [−0.4 to 0.38]	0.961
Within Control change	−2.8 [−7.06 to 1.46]	0.172	−3.4 [−8 to 1.2]	0.129	−0.28 [−0.65 to 0.09]	0.136
Between group difference	−1.64 [−8.55 to 5.27]	0.622	2.73 [−4.54 to 10]	0.439	−0.27 [−0.8 to 0.26]	0.322
Body mass index (kg/m^2^)						
Within ChiRunning change	−0.36 [−0.98 to 0.26]	0.218	−0.54 [−1.45 to 0.37]	0.209	−0.05 [−0.1 to −0.002]	0.042
Within Control change	−0.12 [−0.56 to 0.32]	0.555	−0.05 [−0.42 to 0.31]	0.74	−0.01 [−0.06 to 0.04]	0.726
Between group difference	−0.24 [−0.93 to 0.45]	0.472	−0.49 [−1.35 to 0.38]	0.252	0.04 [−0.03 to 0.11]	0.218

Between group difference is ChiRunning - control

^a^A negative value means a decrease from baseline

^b^Change over time is based on linear mixed model estimates over time. The sign indicates the direction of the slope

In addition, we included n = 4 participants who were taking antihypertensive medication who were all randomly assigned to one of the two control arms of the study. Given that the medication effects could overwhelm the intervention effects, we would not include participants on antihypertensive medications in a larger trial. In this small feasibility study, excluding these participants did not significantly affect differences between the two groups.

In addition to potential systematic bias associated with procedures, the effects on blood pressure in a prehypertensive population are likely to be smaller than in a hypertensive population and therefore may require larger numbers to detect. For example, in a meta-analysis of yoga (another mindful movement-based activity) for blood pressure, authors reported a positive effect for participants with hypertension but mixed findings for prehypertension [21]. Our participants had less room for improvement given their prehypertensive (vs. hypertensive) blood pressures and may require a large study to detect true differences.

### BMI

Changes in BMI between baseline and post intervention and baseline and follow up were in the right direction for both groups (Table 2). While there is no statistical between-group difference in the slope of change over time, only the slope in the ChiRunning group is significantly different from zero (*p* = 0.042), suggesting that a larger study may favor the ChiRunning group for changes in BMI.

Given the sensitivity of blood pressure to external factors (such as time of day or day of the week, at issue in this pilot study) BMI may be a more stable indicator of efficacy and we may see changes in blood pressure with longer follow up. Such a change was seen in a study of 115 prehypertensive participants over 6 months where increased fitness and decreased waist circumference independently predicted a decrease SBP [22].

### Injury

Both the ChiRunning and control group spent similar amounts of time running and had similar proportions of participants with self-reported RRD and self-reported RRI (Table 3). Injury incidence per 100 h of running was also similar between the two groups and similar to what has been found in other studies of novice runners [20, 23]. In the ChiRunning group, the RRI was mostly attributed to a single participant with a previously undiagnosed medical condition aggravated by the increased activity. The participant stopped running but remained enrolled in the study reporting a total of 22 days of RRI and 0 days of RRD. When this participant was excluded from analysis, the average RRI in the ChiRunning group drops by more than than half (ChiRunning 1.9 ± 3.6 vs. control 2.8 ± 2.5, *p* = 0.54).

**Table 3:** Self-reported injury and discomfort over the study for ChiRunning and control groups

	ChiRunning	Control	*P*
	n = 9	n = 10	
Total hours of running, mean ± sd	15.5 ± 7.9	14.4 ± 6.4	0.75
Self-reported running-related injury (RRI)			
Participants reporting RRI, n(%)	5(56)	5(50)	1.0
Days RRI reported, mean ± sd	4.1 ± 7	2.8 ± 3.6	0.61
Incidence of RRI per 100 h run [95 % CI]	4 [1.2 to 8.4]	3 [0.7 to 7.1]	0.72
Self-reported running-related discomfort (RRD)			
Participants reporting RRD, n(%)	7(78)	6(60)	0.63
Days RRD reported, mean ± sd	3.6 ± 4.4	1.9 ± 2.6	0.33
Incidence of RRD per 100 h run [95 % CI]	10 [5.4 to 16.8]	4 [1.5 to 9]	0.06

Running-related injury is discomfort that affected running

Running-related discomfort is discomfort that did not affect running

There was a trend of higher self-reported RRD in the ChiRunning group (*p* = 0.06). This may have been the effect of increased awareness leading to increased sensitivity for RRD, which may play a significant role in injury prevention. In another study, an association between increased age and decreased injury led authors to speculate that age was acting as a proxy for older participants having more experience with “the language of their body” and not necessarily the age of their body per se [24]. Increased body awareness may prevent injury by bringing early attention to bodily cues allowing the individual to take injury prevention measures [25]. Thus, self reporting RRD more frequently is *not necessarily* indicative of a higher injury rate, rather it could indicate a lower threshold for body sensing and by promoting early prevention measures, this sensing may lead to decreased injury over time.

Due to the pilot nature of our study, there were limitations that also need to be considered. One of the objectives was to refine procedures. As part of the intervention refinement, adjustments to the protocol were made throughout the study, and inclusion criteria were revised. Other areas for procedural refinements in a future study were identified, namely a potential bias associated with the timing of blood pressure measurement. In addition, due to the pilot nature of the study, our sample size was small, and thus the study was not powered to detect many significant effects.

## Conclusions

ChiRunning appears to be a feasible and acceptable training technique for people with elevated blood pressure. We did not find that ChiRunning had a significant impact on blood pressure or self-reported injury, but did see a positive affect on BMI. Teaching ChiRunning may be effective for improving blood pressure but warrants further investigation in a larger trial.
